# The bacterial microbiota in first-void urine from men with and without idiopathic urethritis

**DOI:** 10.1371/journal.pone.0201380

**Published:** 2018-07-27

**Authors:** Maria Frølund, Arne Wikström, Peter Lidbrink, Waleed Abu Al-Soud, Niels Larsen, Christoffer Bugge Harder, Søren Johannes Sørensen, Jørgen Skov Jensen, Peter Ahrens

**Affiliations:** 1 Department of Bacteriology, Parasitology and Mycology, Research Unit for Reproductive Tract Microbiology, Statens Serum Institut, Copenhagen, Denmark; 2 Department of Dermato-venereology, Karolinska University Hospital and Institute of Medicine Karolinska Institute, Stockholm, Sweden; 3 Department of Biology, University of Copenhagen, Copenhagen, Denmark; 4 Danish Genome Institute, Aarhus, Denmark; Fred Hutchinson Cancer Research Center, UNITED STATES

## Abstract

**Background:**

Non-gonococcal urethritis (NGU) is a common syndrome in men. NGU may have several causes, but many cases are caused by sexually transmitted infections that may also cause complications in their female partners. *Chlamydia trachomatis* and *Mycoplasma genitalium* are the most common causes of NGU, but in up to 35% of the cases, none of the known viral or bacterial causes are found. Traditionally, pathogens have been detected using various culture techniques that may not identify all species present in the urethra. To address this, we used culture-independent methods for analysis of the male urethral microbiota.

**Methods:**

This case-control study analysed first void urine samples, collected at STD clinics in Stockholm, Sweden from men with idiopathic urethritis (IU), i.e. negative for *Neisseria gonorrhoeae*, *Chlamydia trachomatis*, *Mycoplasma genitalium*, *Ureaplasma urealyticum*, *Trichomonas vaginalis*, adenovirus, and herpes simplex virus type 1 and -2 together with samples from men without urethritis. Forty-six controls and 39 idiopathic urethritis patients were analysed.

**Results:**

The microbiota was highly diverse: None of the 302 operational taxonomic units (OTUs) found in negative controls and IU patients were found in all of the samples or even in all of the samples in one group. More than 50% of the OTUs were only found in one or two of the total of 85 samples. Still the most dominant 1/6 of the genera constituted 79% of the sequences.

Hierarchical clustering in a heatmap showed no specific clustering of patients or controls.

A number of IU patient samples were dominated by a single genus previously related to urethritis (*Gardnerella*, *Haemophilus*, *Ureaplasma*).

**Conclusion:**

The male urethra contain a very diverse composition of bacteria, even in healthy controls. NGU may be caused by a number of different bacteria but more studies including a higher number of samples are needed for elucidation of the role of each species.

## Introduction

Non-gonococcal urethritis (NGU) is a common sexually transmitted infection (STI) in men. In addition to the urethritis symptoms, NGU pathogens may cause complications such as prostatitis and epididymitis [1], and reactive and purulent arthritis [2,3]. By transmission to female partners, the pathogens may cause pelvic inflammatory disease and subsequent infertility[4–6].

*Chlamydia trachomatis*, *Mycoplasma genitalium* and *Trichomonas vaginalis* [7] are well-established causes of NGU. Further, herpes simplex virus type 1 and 2 (HSV-1 and -2)[8], adenovirus [9,10], have been suggested to cause urethritis together with *U*. *urealyticum* when this bacterium is present in high loads [11,12]. However, in up to 35% of NGU cases, none of these known organisms are detected [13] indicating the presence of additional yet unidentified pathogens. It has been a clinical dogma that bladder urine from healthy young men was sterile until it reached the urethra, which is known to be colonised by a variety of bacteria [14]. Traditionally, urine samples have been examined by various culture based techniques that, although well suited for detection of certain pathogens, may overlook other more fastidious bacterial species. Enhanced quantitative techniques, using additional media and incubation conditions have revealed that many bacteria can be cultured from bladder urine even of healthy women [15]. It could be speculated that also in men, more refined identification methods may find hitherto unidentified pathogens in the urine.

Earlier attempts to characterize the uncultivable bacteria of urine and the urethra have included PCR amplification, cloning and Sanger-sequencing of more or less complete 16S rRNA genes [16,17]. This approach gives a very accurate description of the bacteria that are found [18], but the number of sequences that can be handled practically limits the studies to the more abundant bacterial species.

Recent analyses of the human microbiome have shown a rich and diverse microbiota in almost every examined habitat, which could suggest that also the urethra may harbour a more diverse microbiota than previously understood. It has been shown that the microbiota of first-void urine (FVU) samples accurately reflect the microbiota of the distal urethra sampled by obtaining urethral swabs[19]. Thus, in order to characterize the bacterial microbiota of the urethra at a more detailed level and aiming at discovery of new NGU pathogens, we used 454 high-throughput sequencing to analyse urine samples from men with and without urethritis.

## Materials and methods

### Ethics statement

All patients provided oral consent after having received written and oral information regarding the study. The Regional Ethics Committee for Stockholm, Karolinska Institutet, Stockholm, Sweden approved the study (protocol no 2008/695-31/2). Written consent and registration of oral consent was not needed according to the approval of the Ethics Committee.

### Patient samples

The patients were part of an earlier described set of 284 patients from STD clinics in Stockholm, Sweden collected between June 2008 and April 2012[12].

The patient´s age, number of sexual partners within the previous 6 months and number of lifetime sexual partners were recorded as well as urethritis symptoms, antibiotic treatment within the previous 3 months, previous STDs, and information about sexual habits, such as insertive anal- or oral sex within the previous 6 months. No information regarding vaginal sex only and details regarding sexual behaviour at last intercourse was collected.

Urethral smears obtained with a bacteriological loop were examined by microscopy and scored as <5, 5–10, 10–30 and >30 polymorphonuclear leukocytes (PMNL) per high-power (×1000) microscopic field (hpf). Patients were included as IU cases if they were PCR negative for the above mentioned pathogens, had ≥10 PMNL/hpf and urethritis symptoms lasting <30 days. Controls had no urethritis symptoms and <5 PMNL/hpf. Samples with 5–10 PMNL/hpf were discarded. Patients and controls were excluded if they had received any antibiotic treatment within the previous 3 months. From each patient, 7–13 ml of first void urine (FVU) was collected in GeneLock^®^ urine transport tubes (Sierra Molecular, Sonora, CA, USA) and sent to Statens Serum Institut (SSI), Copenhagen by ordinary mail [12]. The collection included samples from men without urethritis (controls) and samples from men with idiopathic urethritis (IU), i.e. those that tested negative for *C*. *trachomatis*, *M*. *genitalium*, *N*. *gonorrhoeae*, *U*. *urealyticum*, *T*. *vaginalis*, HSV-1 and -2, and adenovirus.

### Extraction of DNA from urine samples

For 454-sequencing, bacterial DNA was purified using a modified QIAGEN protocol. In brief, bacteria from 1,900 μl FVU were harvested by centrifugation at 30,000×g for 15 min, re-suspended in 200 μl of 30 mM Tris/HCl pH 8.0; 1 mM EDTA, 15 mg/ml lysozyme, 20μg/ml protease, supplemented with 200 μl AL buffer (QIAGEN, Hilden, Germany) and subjected to bead-beating (0.1 mm zirconium beads, Biospec Products, Bartletsville, OK, USA) at speed setting 7000 for 70s in a MagNA Lyser (Roche). This was followed by 10 min incubation at 56 °C and purification using the DNeasy blood and tissue kit as recommended by the manufacturer (QIAGEN). The DNA was eluted in 200 μl 10 mM Tris/HCl pH 8.5 and stored at –20 °C until 16S PCR for the 454-sequencing was performed.

For the species specific qPCRs, DNA was extracted by boiling the cell pellet with Chelex-100 resin as previously described [20].

### Species specific PCRs

All samples were analysed in specific PCR assays as described earlier [12]. In brief, For *Neisseria gonorrhoeae* we used a slight modification of an earlier published qPCR targeting the *porA* pseudogene[21]. For *C*. *trachomatis* two qPCRs targeting the cryptic plasmid (S1) and the 16S rRNA gene, respectively were used[22], *M*. *genitalium* was identified by a qPCR targeting the *MgPa*-gene[20]. *U*. *urealyticum* and *U*. *parvum* were identified in a multiplex qPCR targeting the urease gene[23]. *T*. *vaginalis* was identified with a conventional PCR which amplifies a part of a repeat [24]. For Adenovirus a qPCR amplifying the hexon gene was used[25], finally, HSV -1 and -2 were detected by an unpublished multiplex qPCR, amplifying parts of the G and D gene respectively (for details see [26]).

#### Broad-range 16S qPCR for quantification of the total bacterial load

To determine the bacterial load in the extracted samples, quantitative broad range 16S rDNA PCR, amplifying the V3–V4 region, was performed as described [27], modified from [28]. Five μl of the sample was used as template. PCR-grade water was used as negative controls in duplicate wells in all 96-well plates.

### Sequencing

The V3 and V4 regions of the 16S rRNA gene were amplified by PCR with primers MPRK341F (5’-CCTACGGGRBGCASCAG-3’) and MPRK806R (5’-GGACTACNNGGGTATCTAAT-3’) [29].

The amplification was done in triplicate on three separate days in 25μl of 1×Phusion^®^ HF buffer containing 0.2 mM dNTP mixture, 0.5 U Phusion^®^ Hot Start High-Fidelity DNA Polymerase (Finnzymes Oy, Espoo, Finland), 0.5 μM of each primer and 2 μl template DNA. PCR conditions were 30 sec at 98°C followed by 30 cycles of 5 sec at 98°C, 20 sec at 56°C and 20 sec at 72°C followed by a final extension for 5 min at 72°C. All amplifications included positive (*Legionella*) and negative (H_2_O) amplification controls. Most amplification controls were negative and not processed further, one positive amplification control sample was included in the sequencing. The amplicons were separated on 1% agarose gels, excised and purified with QIAEX II Gel Extraction Kit (QIAGEN) as recommended by the supplier, and after adjusting the concentration for each amplicon, the triplicates were pooled in equimolar amounts. The purified PCR products were tagged by another round of PCR (15 cycles) using primers 341F and 806R with adapters and tags (MIDs) as previously described [30]. The tagged PCR amplicons were gel purified as described above, quantified using a Qubit^®^ Fluorometer (Invitrogen) and diluted to approximately 4×10^5^ copies per μl. Approximately 30 samples per run were sequenced on one of two regions of a 70×75 GS FLX Titanium PicoTiterPlate (PTP) using a GS FLX Titanium pyrosequencing system according to the manufacturer’s instructions (Roche).

### Sequence analysis

Any contaminating human or chloroplast sequences were removed using the Bowtie software [31] and the (hg19) version of the human genome sequence and a database of the 1490 chloroplast sequences from the Ribosomal Database Project (RDP) version 11.04[32,33]. For further elimination of possible contaminants, genera that were identified as purification kit contaminants by Salter et al [34] were removed. Also OTUs with a relative abundance that was significantly inversely related to the total bacterial DNA load were removed[35,36].

The 16S rRNA gene sequences were analysed using BION-meta software package version 16.3[37]. After demultiplexing, quality and primer trimming, sequences shorter than 250 bp were removed. Following chimera-filtering, where scores > 25 were removed, the remaining sequences were mapped against the database “RDP_SSU_340-807-All”, a position 340 to 807 slice of the aligned RDP-SSU database version 11.04. Taxonomic classification was based on a similarity of 97%. Taxons that constituted less than 10 sequences in a sample were omitted. For quantitative comparisons sequence number in each sample was normalized to lowest number of sequences.

### Statistics

Fisher´s exact test for dichotomous variables. The Mann-Whitney test was used for continuous variables when comparing two groups. Spearman´s rank correlation was used to calculate the correlation between patient characteristic and 454-sequencing. All qPCR results are presented as genome equivalents per ml original urine sample. Significance level was 5% and two-sided results with adjustment for multiple comparisons were used throughout. Pearson correlation using Benjamini-Hochberg adjustment[38] for false discovery rate was used for analysis of possible contaminants.

The R program package version 3.0.2 was used for ecological analysis and heatmaps (The R Foundation for Statistical Computing, Vienna, Austria)[39]. For heatmaps, we used the heatmap.2 function from the gplots package with Euclidian distance.

For calculation of alpha-diversity and Bray-Curtis dissimilarity we used the Vegan package[40].

## Results

### Samples

**Fig 1:**
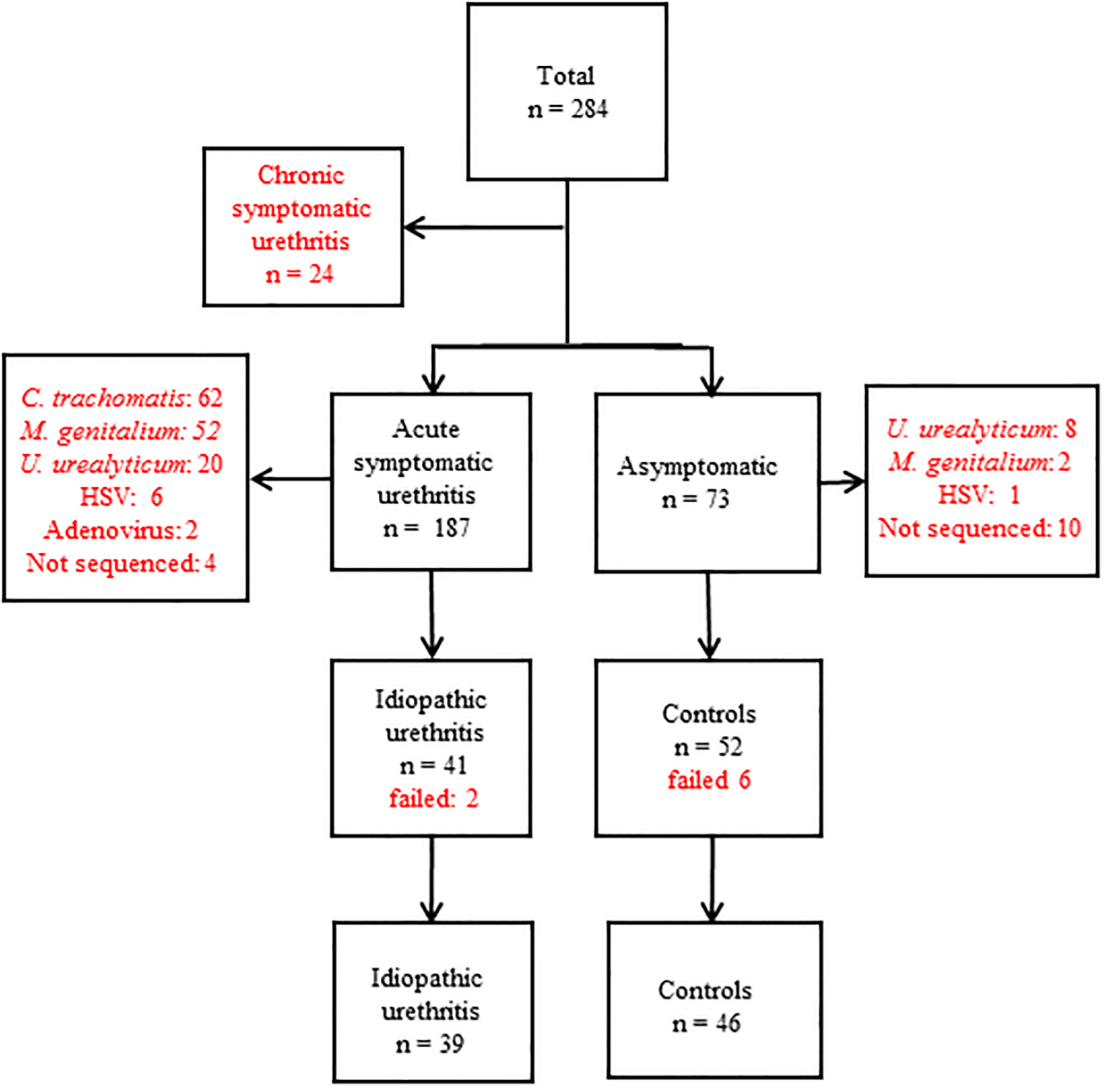
Flow-chart showing inclusion of first void urine samples from patients and controls for 454-sequencing. Excluded samples are shown in red. A total of 93 samples were sequenced. A total of 85 samples were subsequently used in the analyses.

From a total of 284 patients and controls, all samples from patients with chronic symptomatic urethritis, or samples that were postitive for *C*. *trachomatis*, *M*, *genitalium*, *U*. *urealyticum*, Herpes virus or Adenovirus were excluded. Further 14 samples, four from patients and 10 from controls were not sequenced due to insufficient amounts of DNA in the samples. Of the 93 samples that were sequenced by 454-pyrosequencing; 52 were from men without urethritis (controls) and 41 were from men with acute IU. Eight samples were excluded as the sequences were few or of poor quality (Fig 1). The remaining 85 samples were used for taxonomical classification.

**Fig 2:**
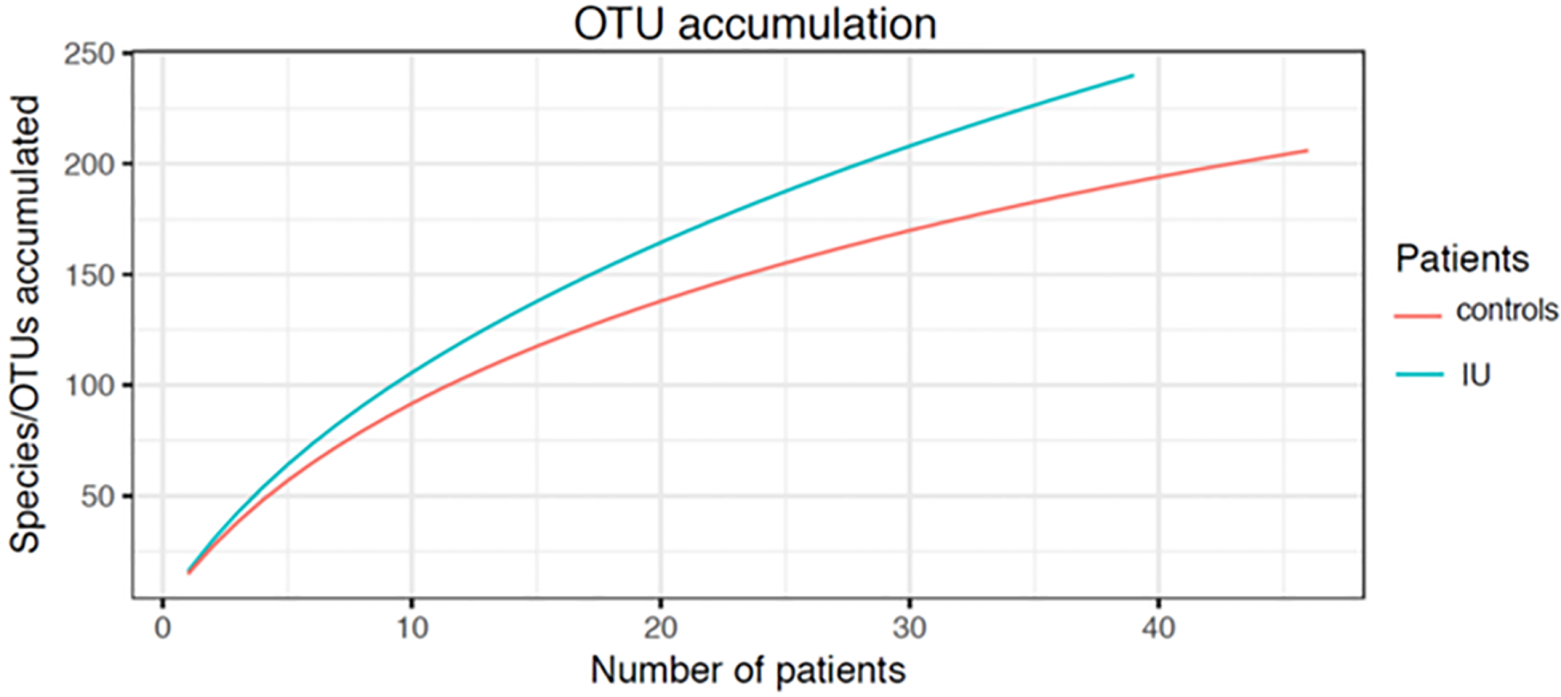
OTU accumulation curves for 39 IU patients (blue) and 46 controls (red).

Rarefaction analysis of the individual samples (supplementary data S1 Fig) indicated that all curves reached a plateau suggesting that a sufficient number of sequences had been analyzed. However, the distribution of taxons varied considerably between samples. Species accumulation curves of the combined IU or negative control samples suggested that even 85 patients and controls were not sufficient to describe the complete male urethral microbiota (Fig 2).

While the overall average alpha-diversity (Fisher) did not differ much between patients (2.50) and controls (2.60), (t-test, unequal variances, p = 0.078), the bacterial community composition of the patient group was, on average, both significantly more uneven and heterogeneous; Pielou´s evenness was 0.78 and 0.65 for patients and controls, respectively (t-test, unequal variance, p = 0.009). The average pairwise (Bray-Curtis) dissimilarity within each group was (0.92) for the patients compared to 0.9 in the control group, which was slightly but significantly higher (t-test, equal variance, p<0.00064). See supplementary data S2 Fig).

### Patient characteristics and species-specific qPCR results

After sequence filtering, 39 men with IU and 46 controls without urethritis were left for further analysis. Patients´ age, number of sexual partners within the last six months or lifetime, sexual habits i.e insertive anal sex or receiving oral sex, did not differ between patients with IU and controls. Only two men had sex with men and were not analysed separately. Men with IU reported previous STDs such as “other NGU” (11 of 38 (28%)) and chlamydia (17 of 38 (44%)) more often than controls (4 of 45 (9%)) and (7 of 46 (15%)), (“other NGU”, p = 0.002 and chlamydia, p = 0.007 comparing IU and controls), respectively (Table 1). As patients attending the clinic for condyloma treatment were also included as controls, they appeared with a higher frequency in the control group compared to the IU group (p = 0.008).

**Table 1:** Characteristics of 46 men without NGU and 39 men with idiopathic urethritis.

Characteristic	Controls without NGU (n = 46)	Acute symptomatic idiopathic urehritis^a^ (n = 39)	
Age in years	28 (19–51)	30 (20–52)	ns
No. sex partners, past six months	2 (0–15)	2 (1–10)^b^	ns
No. life-time sex partners	25 (2–150)^d^	20 (3–150)^c^	ns
Symptoms			
Duration (days)	–	7 (1–28)	
Dysuria	–	34 (87)	
Objective discharge	–	33 (85)	
Subjective discharge	–	19 (49)	
Oral sex (y/n)^d^	18/5	13/7	ns
Anal sex (y/n)^d^	4/19	2/17	ns
MSM PMNL/hpf	1	1	
<5	46	–	
10–30	–	23 (59)	
>30	–	16 (41)	
Previous STD			
*C*. *trachomatis*	7 (16)	17 (45)	p = 0.007
*M*. *genitalium*	1 (2)	1 (3)	ns
*N*. *gonorrhoeae*	1 (2)	2 (5)	ns
HSV	2 (4)	0 (0)	ns
Condyloma	20 (44)	6 (16)	p = 0.008
Other NGU	4 (9)	11 (29)	p = 0.0023
Never	17 (38)	9 (24)	ns
ND	1	1	ns

Continuous variables are summarized as median (range) and categorical variables as frequency (%).

Idiopathic urethritis patients and controls were negative for *C*. *trachomatis*, *M*. *genitalium*, *N*. *gonorrhoeae*, *U*. *urealyticum*, *T*. *vaginalis*, HSV-1 and -2, and adenovirus. ns = not significant

^a^ Urethritis symptoms 1–30 days.

^b^ No information for 2 patients.

^c^ No information for 5 patients.

^d^ Patient information obtained only for the “y/n” in the table. Y; yes, N; no

Fisher´s exact test was used for dichotomous variables. The Mann-Whitney test was used for continuous variables when comparing two groups.

### The total bacterial load determined by 16S qPCR

The total bacterial 16S rDNA load was determined in 83 of the 85 included samples. Two samples did not contain sufficient DNA for analysis. The approximate limit of detection was 100 copies /ml of FVU [27]. The median bacterial load was 8.0×10^3^ geq/ml urine (range 2.1×10^2^–2.1×10^5^ geq/ml) in the controls, 9.2×10^3^ geq/ml (range 5.3×10^2^–2.1×10^8^ geq/ml) in the IU patients, and 8.4×10^3^ geq/ml (range 2.0×10^3^–6.1×10^4^ geq/ml). The bacterial loads determined by 16S qPCR did not differ statistically between the patient groups. One control and was not examined in the 16S qPCR assay due to lack of sample material.

### Distribution of sequences and taxonomic units

Sequences from the 39 patients with IU and the 46 controls were classified according to RDP taxonomy.

The bacterial load was also used to identify possible contaminants. Using Pearson correlation the taxons *Propionibacterium*, *Staphylococcus*, *Corynebacterium*, *Acinetobacter*. *Streptococcus*, *Micrococcus*, *Comamonas*, *Escherichia*, *Paracoccus*, *Arthrobacter* and an unclassified Bacterial OUT were found significantly negatively associated with DNA concentration in the samples. These taxons were considered contaminants and were removed from the analysis.

The total number of unique OTU´s at the, class-, order-, family- and genus- levels were 38, 63, 116 and 302 respectively. The quality of the NGS sequences did not allow a general classification to the species level. A large part of the microbiota in the IU patients and controls without NGU was shared at all taxonomic levels; e.g. at the genus level, 67% of the total unique OTU´s were present in the controls and 78% in the IU patients. The single no-template control sample that turned out positive in the PCR contained bacterial DNA.

#### Composition of the urine microbiota at the genus level in IU patients and controls

Patient characteristics were compared for individual taxa. However, we found no significant correlation between taxa and patients age, number of sexual partners the last 6 months or lifetime partners and previous or current condyloma. Nor was there significant correlation to oral sex or to the duration of symptoms. Finally we did not observe any different distribution of taxons between patients with 10–30 PMNL and patients with > 30 PMNL.

The 39 IU and 46 controls contained a total of 302 different genera; 201 (67%) of them were found in the controls and 235 (78%) in the IU patients. The distribution of genera varied considerably between samples. No genus was present in all samples or even in all samples from patients or from the controls. In the controls, two genera, *Lactobacillus* and an unclassified *Alphaproteobacterium*, were present in 50% and 71% of the samples, respectively, while only 11 of the 201 genera were present in more than 25% of the samples. In IU patients 0 and 15 of the 235 genera were found in >50% or >25% of the samples, respectively. Nine (82%) the 11 genera found in >25% of the controls were also found in >25% of the IU patients. At the genus level, the median number of different OTUs in IU patients was 17 (1–51) and 16.5 (4–28) in the controls. A total of 176 (58%) genera were only present in one or two samples (126(42%) in only one, 50 (17%) in two samples). A complete list of taxons at genus level found in this study is shown in supplementary S1 Table.

**Fig 3:**
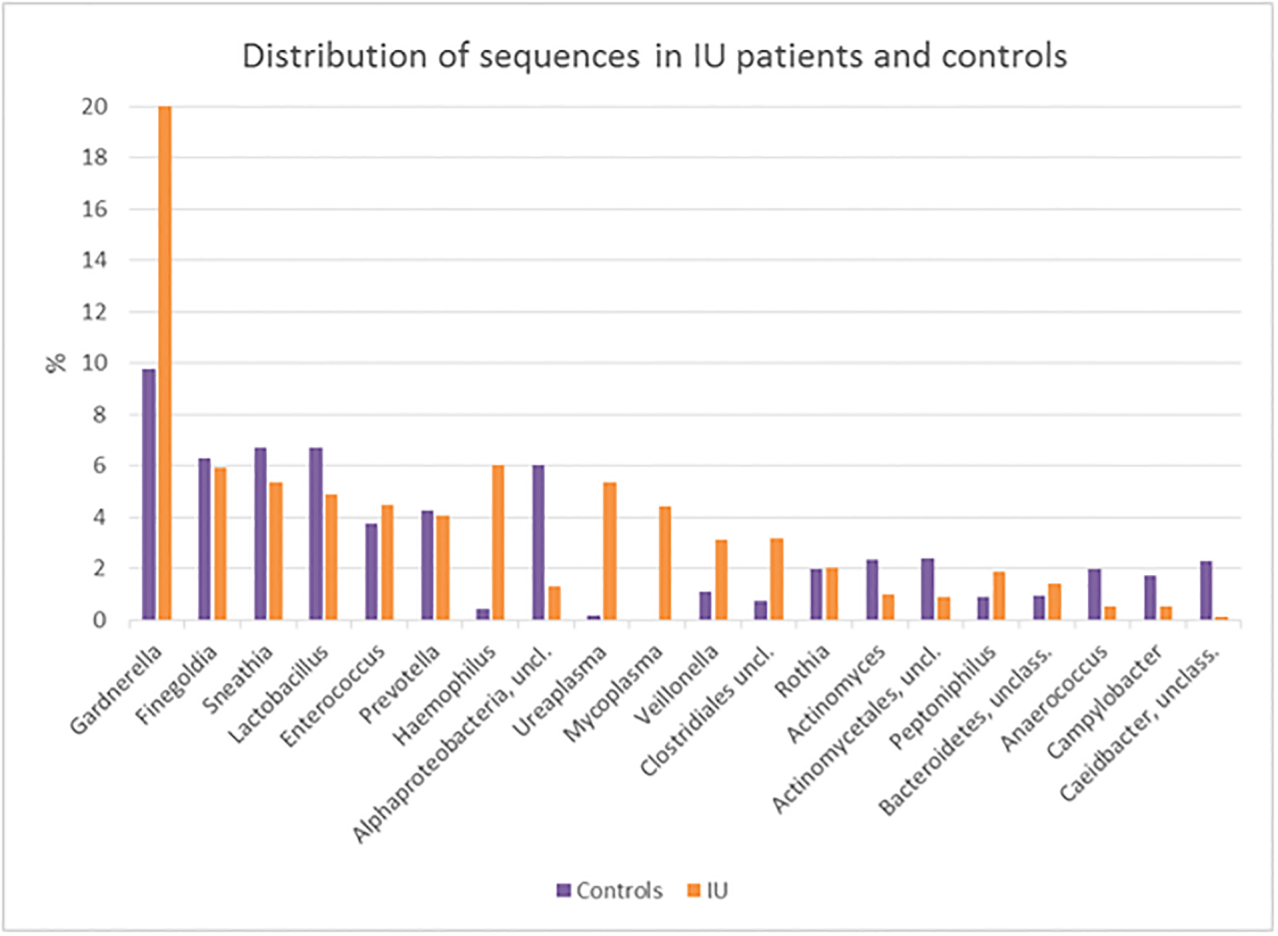
Distribution of sequences (% of total) in all controls (purple) and all IU patients (orange) for the 20 genera each constituting more than 1% of the total number of sequences.

The distribution of reads from the 20 most prevalent OTUs at the genus level and present as >1% of the total number of sequences in controls and in IU patients is displayed in Fig 3. These 20 OTUs represented 70% of total number of sequences.

*Mycoplasma* spp. was only found in the IU patients. For the taxons *Haemophilus*, *Ureaplasma*, and unclassified *Clostridiales*, the presence in IU vs controls was more than 4-fold higher. The opposite ratio was found for *Alphaproteobacterium* and *Caedibacter*.

**Fig 4:**
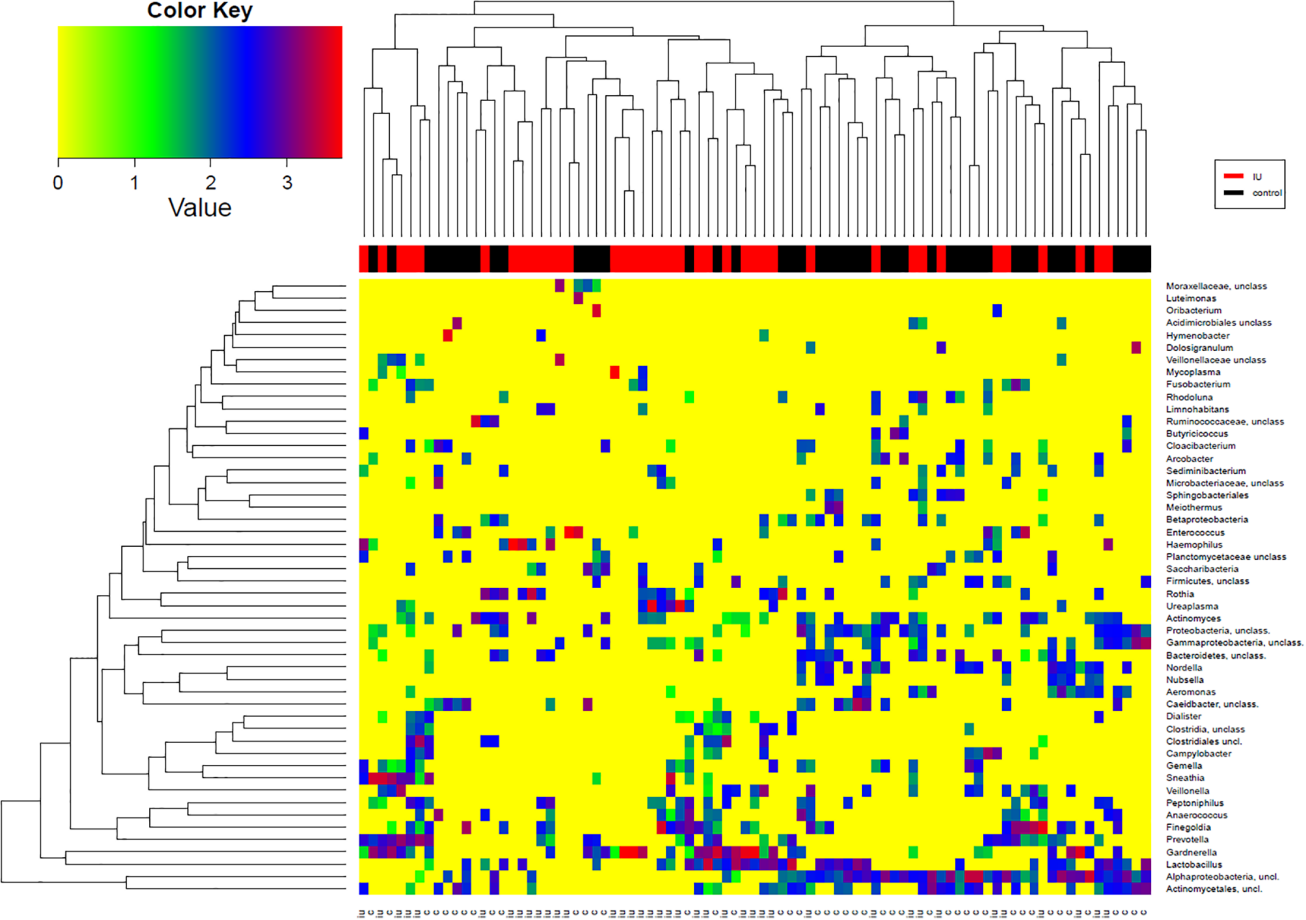
Heat map of the 50 dominant genera in 39 patients with idiopathic urethritis and 46 healthy controls. Colour Key values are logarithmic values. IU; idiopathic urethritis.

To visualize the associations between the microbiota in IU patients and controls at the genus level, a heatmap of the 50 most dominant genera is shown in Fig 4. These 50 genera (comprising 17% of all genera) constituted 79% of all sequences and were present in a median of 12 samples (range 2–46). The diversity between individuals was evident in both patients and controls. There was no strong grouping of patients versus controls, and all clusters contained samples from both groups. By mere presence, three genera were significantly differently distributed: *Alphaproteobacteria* were found more frequent in the controls than in IU patients (33 (72%) positive vs 13 (33%) positive (p = 0.0005)). *Ureaplasma* and *Mycoplasma* were found more frequent in IU patients than controls (8 positive (21%) vs 2 positive (4%) (p<0.04)) and 4 positive (10%) vs 0 positive (0%) (p<0.05) respectively.

**Fig 5:**
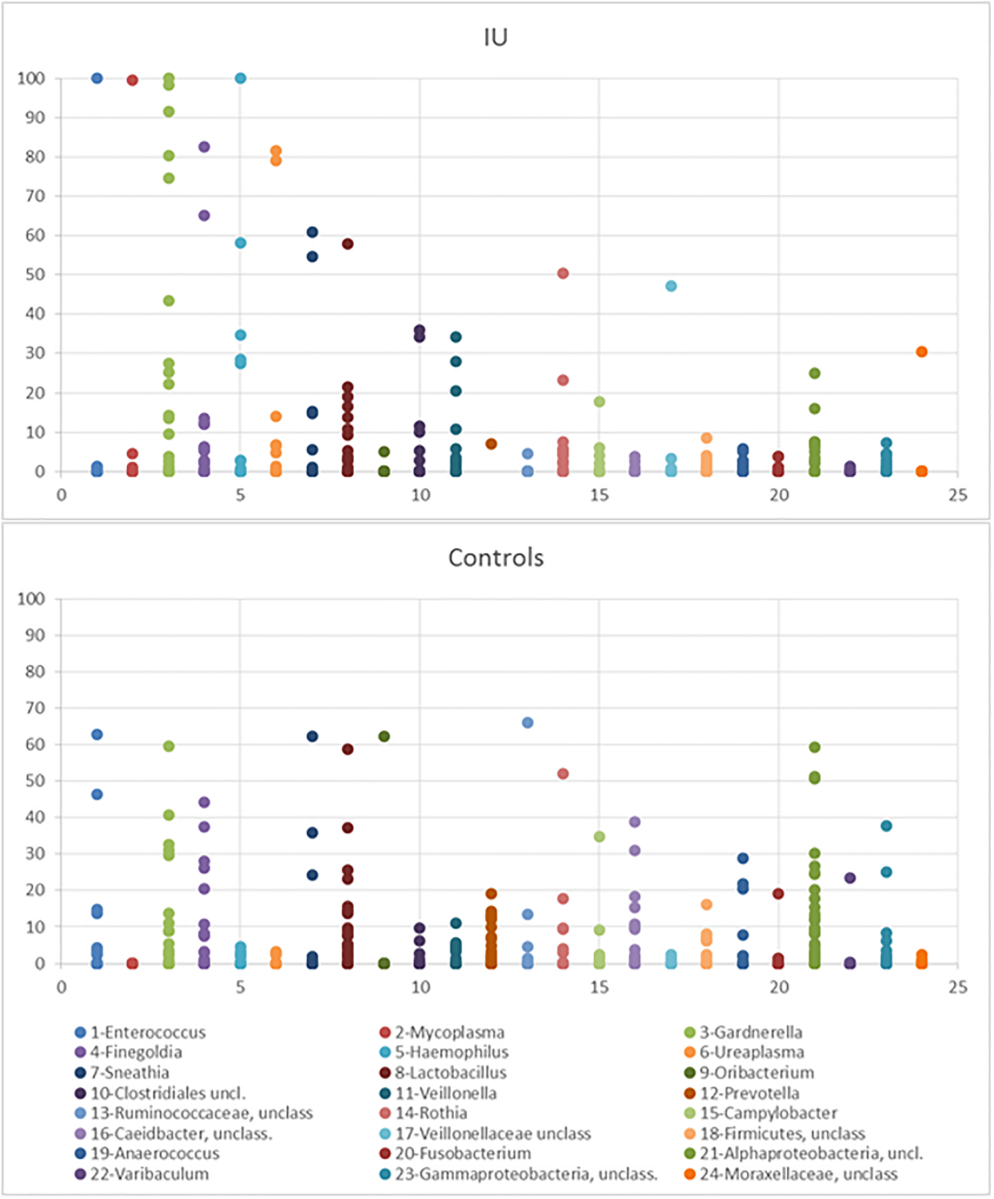
Presence (%) of the 24 most dominant OTUs in individual samples. Top panel patients with idiopathic urethritis, bottom control.

The relative proportion of reads for the 24 most dominant OTUs in individual samples is shown in Fig 5. For each of the 24 genera, the representation (0–100%) in the individual samples is shown. For 11 of the 39 IU patient samples (28%), a single genus constituted more than 70% of the sequences while this was not found in any sample (0%) in the control group (p = 0.0001).

The dominant genera in IU patients were *Enterococcus* (one sample), *Mycoplasma* (one sample), *Gardnerella* (five samples), *Finegoldia* (one sample), *Haemophilus* (one sample) and *Ureaplasma* (*parvum*)(two samples).

*U*. *urealyticum* and *U*. *parvum* could not be distinguished on the basis of the 16 sequence used here, but all of the samples found *Ureaplasma urealyticum* positive in species-specific PCR were excluded. By qPCR we found 10/46 controls and 9/39 iu patients *U parvum* positive (ns). Of those positive, the median and mean was higher in patients as compared to controls, but not significant. A BLAST analysis of the *Mycoplasma* sequences found dominant in one sample showed it to be *M*. *penetrans*. *Mycoplasma hominis* was also found in low numbers in three other IU patients.

## Discussion

In this case-control study we used 454 sequencing of 16S rRNA gene amplicons to characterize the bacterial microbiota in urine from men with and without idiopathic urethritis. The approach was used to search for new bacterial pathogens in NGU, as approximately 35% of cases remains unexplained after excluding already established aetiologies. We found the urethral microbiota highly complex, both in patients and in healthy controls.

Urethritis is conventionally defined by the presence of >4 PMNL/hpf in a urethral smear as a cut-off, although the recent US STD treatment guidelines suggest the cut-off to be lowered to *≥* 2 PMNL/hpf in the presence of symptoms[41] [42], however, the cut-off is highly dependent on the sample collection method [43]. In the present study, we aimed to increase the specificity of the IU diagnosis by increasing the cut-off to ≥10 PMNL/hpf and avoiding the “grey-zone positive” NGU with 5–9 PMNL/hpf. This cut-off is supported by a study from [44], which suggested that using a blunt curette for obtaining the sample, an extremely low proportion of chlamydia, gonorrhoeae, and *M*. *genitalium* cases were found in the <5 PMNL stratum. The study also demonstrated that approximately 85% of all NGU caused by *C*. *trachomatis* or *M*. *genitalium* was found in men with ≥10 PMNL/hpf. As samples for microscopy in the present study were obtained with a microbiological loop assumed to have the same sensitivity as the blunt curette, we find it unlikely that using the *≥* 2 PMNL/hpf cut-off suggested by the CDC STD guideline, would have significantly reduced selection bias. Obviously, the findings of the present study will relate only to the PMNL groupings described here and may not be generalizable to other definitions of urethritis. However, the aim of the study was to identify new pathogens and, should such be detected, a larger study on an independent sample set using specific qPCR primers to confirm the findings was planned.

A significantly higher proportion of the men in the control group had condyloma. Given the possible effects of HPV infection on the female vaginal microbiota[45] this could theoretically affect also the male urethral microbiota. However, as very few of the patients attending for condyloma treatment had intraurethral condyloma, this effect could not be studied.

Traditionally, urine of healthy individuals has been considered sterile [46], but more recent studies have found that even the urinary tract of healthy persons harbors a microbiota (reviewed by Whiteside *et al*)[47]. A straightforward hypothesis would be that urethritis patients have a higher bacterial load in the urethra as compared to controls without clinical signs, if the cause of the condition were bacterial. However, the median load of 16S rRNA genes, as determined by broad-range qPCR, was approximately the same in the two groups. Earlier studies have found higher loads of specific pathogens in patients as compared to controls [11,25,48], but these studies used species specific PCRs and did not include determination of the total bacterial load in the urethra. Our findings suggest that male urine may contain a relatively stable amount of bacteria, irrespective of inflammatory status. This is not surprising, as e.g. *M*. *genitalium* urethritis can be caused by even very low numbers of the bacterium [20,49] and this could be true for other pathogens of the urethra as well. Bacteria in first void urine is a combination of the microbiota of the bladder, the urethra, and from the skin and other contact environment. In this combined pool, even substantial disease-related fluctuations in the urethral microbiota may be obscured by other parts the microbiota. On the other hand, Dong *et al* [19] found the microbiota of paired swab-samples and urine samples to be very similar, also quantitatively, suggesting that background microbiota may be of less importance.

The urine microbiota has been examined using next generation sequencing in other studies [19,50–52], but most of these studies used mid-stream or catheterized urine samples, thus minimising the influence of the urethral microbiota. To our knowledge, the present study is the first case-control study of urethritis patients using NGS in this scale. The few previous studies using NGS to characterize the urine and urethral microbiota in asymptomatic men without NGU [50,53] [19] have revealed a diverse bacterial microbiota, highly variable from person to person. The present study confirms this high diversity, as the 39 IU patients and 46 controls in the analysis harboured 302 different taxons, including yet unclassified ones. Further, rarefaction analysis suggested that even more taxons could be part of the male urethral microbiota. Other studies have found fewer genera. Dong et al [19] found 88 genera in samples from10 STI-positive men (*C*. *trachomatis*, *N*. *gonorrhoeae* or *T*. *vaginalis*), and 131 genera in the 22 STI negative men which was approximately 75% of the number of taxons we found in a similar number of samples.

Lewis *et al* [50] also found relatively few genera in 6 patients, but with most patients of a different age-group, than those in our study. These studies have used other patient groups and other experimental settings and are therefore difficult to compare. Furthermore, both of these earlier studies used another region of the rRNA gene (V1-V3) as compared(V3-V4) to the present study, and these different variable regions may possess different capability to classify the individual sequences and a different selectivity of the primers has also been shown [54].

In a study of first void urine, using the 16S ribosomal RNA gene based clone library method, You *et al* found 95 phylotypes in 2427 clones from 38 patients[18]. In comparison with 454-sequencing, the clone library method of You *et al* includes slightly longer amplicons and Sanger sequencing, which is often of better quality than sequence obtained by 454 pyrosequencing. However, much fewer sequences per sample were obtained. Though the methods are not directly comparable, it is noteworthy that >60% of the genera found in that study, were also found in the present study. This high number is perhaps surprising considering the different methods used and the fact that more than 50% of the genera found in our study, were present in only one or two of 96 specimens.

An inherent weakness of this type of 16S sequence-based analysis is the normalizations performed. The initial PCR itself has some normalizing effect, giving relatively less amplification to samples with higher bacterial load. Further, an equal amount of PCR-product from each sample was used for sequencing, whereby all samples, should return the same number of sequences irrespective of the original bacterial load. This may lead to an overestimation of the background microbiota in samples with low DNA yield, as compared to samples with more bacteria and a higher DNA concentration. Thus, the true distribution of taxons present in a given environment can be difficult to establish.

One of the purposes of this study was to search for hitherto unknown bacterial causes of urethritis. Idiopathic urethritis is likely to be caused by multiple different factors, including different bacterial species. *Alphaproteobacteria* was found significantly more frequent in controls. This could perhaps suggest a probiotic role of this bacterium, but more studies, including proper identification, are needed here. *Mycoplasma* and *Ureaplasma* were found more often in the IU patients, but only just at the limit of significance. The *Mycoplasma* included one sample with high level of *M*. *fermentans* and three samples with low level of *M*. *hominis*. However, analyzed separately, the presence of these species was not significantly higher in patients than controls. High level of *U*. *parvum* have in earlier studies been found associated with urethritis [48,55], but low level of *U*. *parvum*, is generally considered insignificant. The inability to identify new genera associated with urethritis could be due to too few samples included to reach sufficient power of the study. Indeed, a power analysis suggested that with 46 controls and 39 IU cases, an IU pathogen would need to be present in 30% of the IU cases compared to 5% among the controls to detect a significant difference at the 5% level with a power of 80% and this is even without taking correction for multiple comparisons into consideration.

In spite of considerable differences, a heatmap of the 50 more prominent OTUs did not show distinct clustering of patients versus controls (Fig 4). This could be due to the large individual variation that we observed. Also there was no evident clustering of the fifty most dominant genera.

We found a number of samples dominated by a single genus. Though most individual taxa were too few for statistical analysis, the dominance of one taxon appears more frequent in IU patients than in controls. A number of these dominant taxa have earlier been described associated with urethritis. This could suggest that a broad spectrum of bacteria can cause IUs (Fig 5).

In 5/39 of the IU samples *Gardnerella* constituted over 70% of the sequences. *Gardnerella* is mainly described in association with bacterial vaginosis in women, but has been suggested as a cause of urethritis in men [56,57], while another study by Bradshaw *et al* could not support this [8]. Indeed, Keane *et al* [58] found a strong correlation between BV and NGU in the male partner with 71% of male partners to women with BV having urethritis as compared with 33% of the partners of BV negative women. The correlations was also inverse with 31% of female partners to men with NGU having BV as compared to 8% of partners without NGU. However, as several STIs are correlated with BV[59], the association is complex. Along the same line, Manhart *et al* found *Sneathia* spp. which is commonly present in BV to be associated with male IU [60]. This study, however, did not examine for *G*. *vagnialis* and a large proportion of the cases were of black ethnicity which may be of importance as black women have a significantly higher prevalence of BV as compared to Caucasian women. You *et al*[18] found *Gardnerella* as the predominant species in 3/38 Japanese urethritis patients analyzed with the 16s clone library method. Interpretation of such smaller findings is difficult. The *Gardnerella* found in male urine could represent temporary passive transfer from their female sexual partners, or could represent true colonization of the male urethra. However, the fact that *Gardnerella* was found as the dominant genus, favors the view that some kind of colonization has happened; otherwise, we would have expected to see much more *Lactobacillus* passively transferred in other men, as this genus is by far the dominant bacterial genus found in the vagina. A direct role for *Gardnerella* in relation to male urethritis thus still awaits further elucidation.

*Haemophilus* constituted almost all of the sequences in one sample, but also more than 25% in another 4 samples. In the parent study (12), *H*. *influenzae* was detected only in men with acute NGU, but only in 5% of these and not statistically significant. Deza *et al* [61] isolated *Haemophilus*, mostly *H*. *parainfluenzae*, but also *H*. *influenzae*, from 12.1% of men with acute urethritis, mainly MSM. In 6.8% of the cases, they isolated *Haemophilus* as the single pathogen, suggesting that members of this genus could be the cause of urethritis. Further, these *Haemophilus*-positive patients all reported unprotected oral sex, indicating a possible route of infection. Bradshaw *et al* [8] suggested that unprotected insertive oral sex may be a mode of transmission and Barbee *et al* [62] estimated that up to 1/3 of NCVNGU in MSM is orally acquired. Thus, bacteria from the oral cavity may play a role in urethritis but more elaborate studies are needed. Unfortunately we only obtained information on oral sex for two (both positive) of the five patients with elevated levels of *Haemophilus*.

More than 75% of the sequences in two samples were *Ureaplasma parvum*. In contrast to *U*. *urealyticum*, *U*. *parvum* has generally been considered of less importance in male acute NGU. However, a few studies have found high loads of *U*. *parvum* related to urethritis [48,55] and in the parent study, this species was associated with chronic, but not acute NGU (12).

*Enterococcus* is a common cause of urinary tract infection but a relation to urethritis in men has not been described. The finding of one patient with >99% *Enterococcus* reads could represent a urinary tract infection often leading also to symptoms and signs of urethritis.

*Mycoplasma penetrans* has earlier been found in urine but not linked to urethritis. Deguchi *et al* were unable to find *M*. *penetrans* in any of 108 men with acute non gonococcal urethritis [63]. Chen *et al* [64] found 1.6% of 1541 HIV/AIDS patients positive for *M*. *penetrans*. Unfortunately, the HIV status of the single patient with highly dominating *M*. *penetrans* was not known.

*Finegoldia* (*peptostreptococcus*) is not described as causes of male urethritis, but is commonly found in the vagina [65]. *Finegoldia* is slightly more common in BV, but also found in 41% of women with normal Nugent scores [66]. In our data *Finegoldia* appears with almost the same frequency as *Lactobacillus*, another common vaginal inhabitant (Fig 2). These latter findings may simply reflect recent unprotected vaginal intercourse.

In conclusion, urine from men with and without idiopathic urethritis as described by 454-pyrosequencing of 16S rRNA amplicons was shown to contain a very diverse microbiota. While more studies are needed, we find no clear evidence for a “core” microbiota. Even if such a core microbiota should exist in healthy individuals, our results suggest that it can be completely disturbed by certain urethra infections. Approximately 1/3 of the IU cases contained a high abundance of different bacteria that have earlier been linked to urethritis though each of these were too few for statistical significance.

## Supporting information

S1 Fig IU_rarefaction. Species accumulation curves for 39 IU patients.
(PDF)

S2 Fig Species accumulation curves for 46 controls. (PDF)

S3 Fig Bray-Curtis. Bray-Curtis dissimilarity for 46 Controls and 39 IU patients.
(PDF)

S4 Fig Bacterial loads. Bacterial load in IU patients and controls as determined by qPCR.
(TIF)

S1 Table Genera in IU patients and controls. Distribution of genera in 39 IU patients and 46 controls.
(XLSX)
