# Supplementary figures and images for: The bacterial microbiota in first-void urine from men with and without idiopathic urethritis
Source: PLoS One. 2018 Jul 27;13(7):e0201380. doi: 10.1371/journal.pone.0201380 (PMC6063444; doi:10.1371/journal.pone.0201380)

# IU, rarefaction(raw samples)

OTUs in sample

Sequences in sample

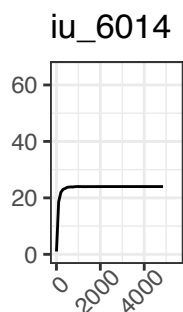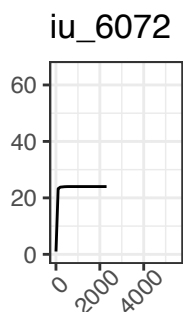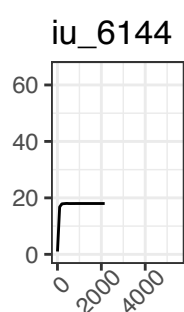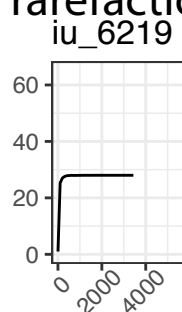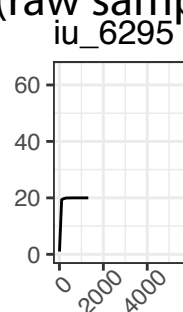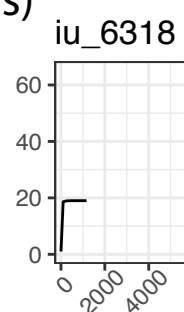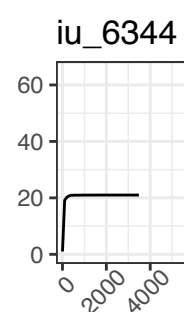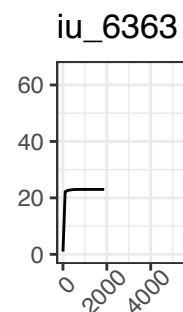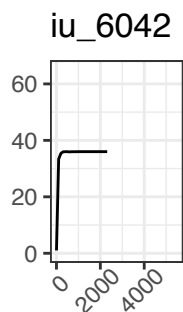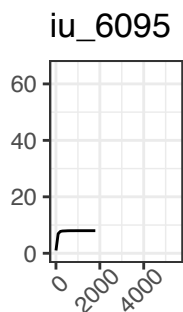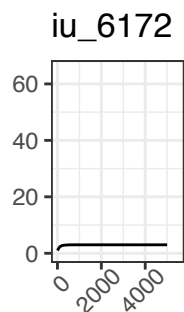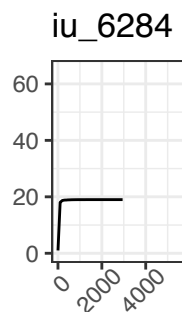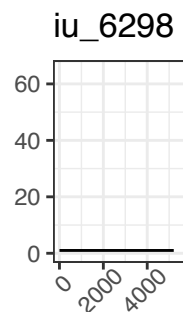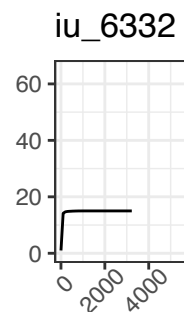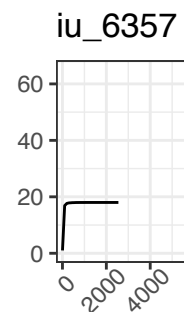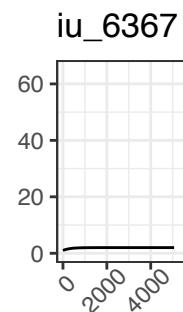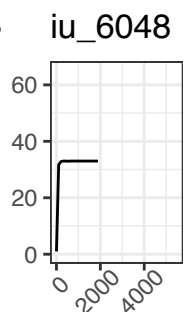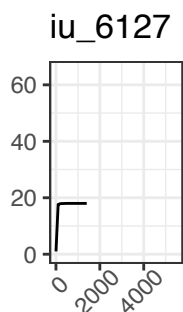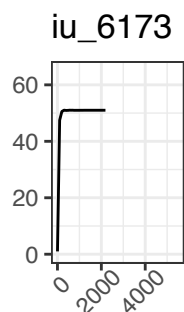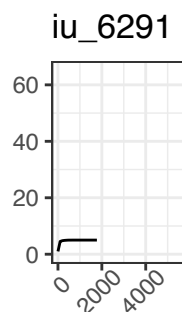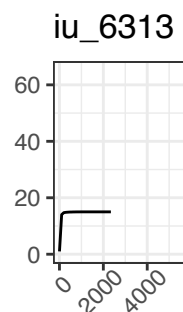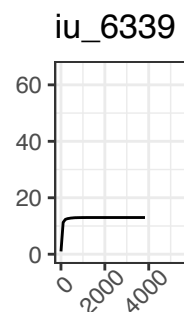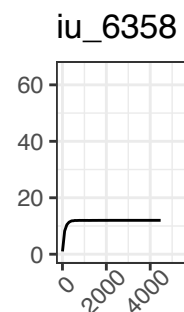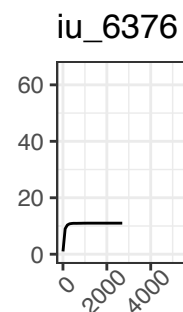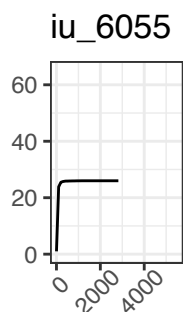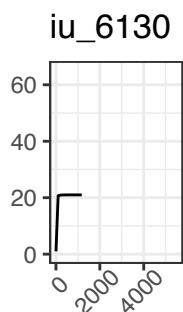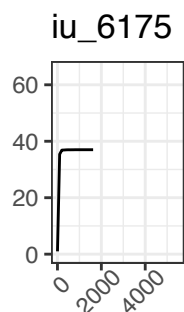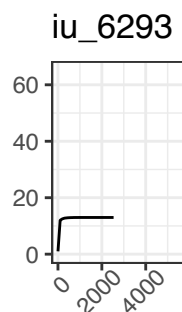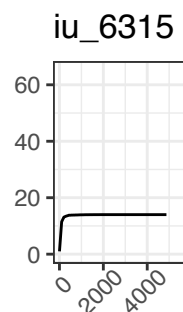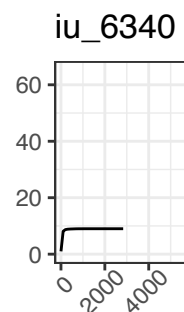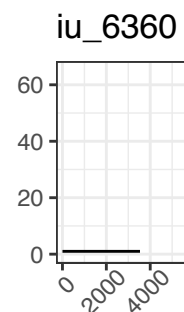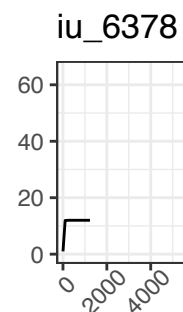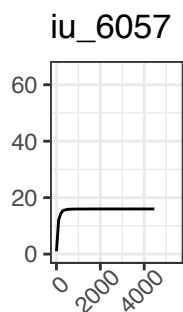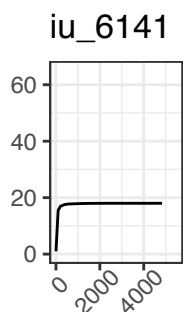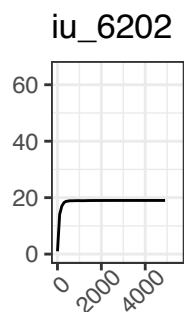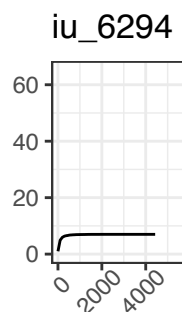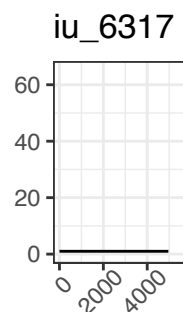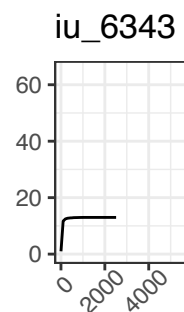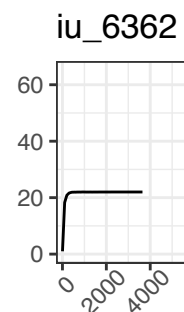

Supplement: S1 Fig — Species accumulation curves for 39 IU patients. (PDF) [file pone.0201380.s001.pdf]

# Controls, rarefaction(raw samples)

OTUs in sample

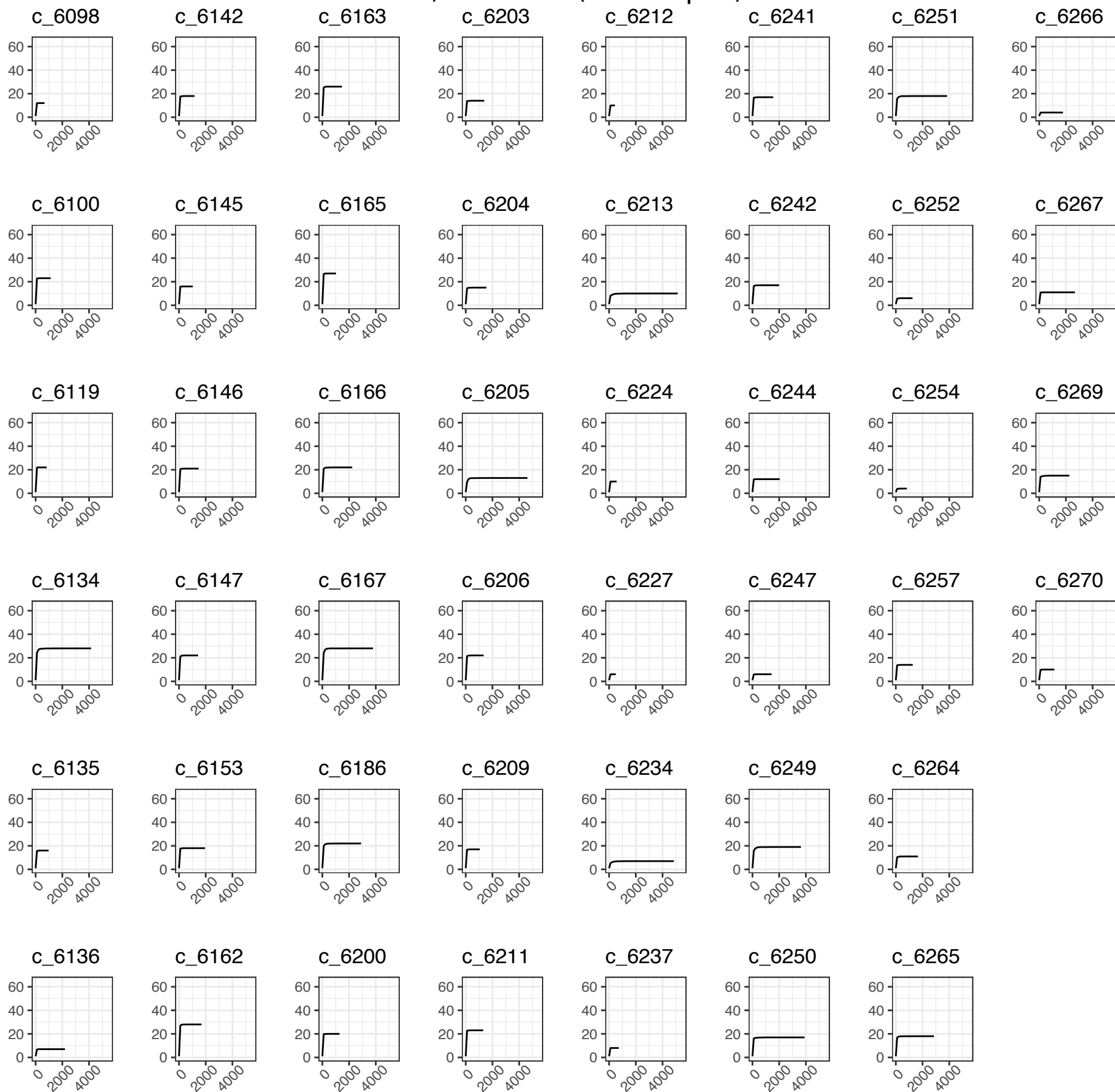

Sequences in sample

Supplement: S2 Fig — (PDF) [file pone.0201380.s002.pdf]

# Bray-Curtis dissimilarity

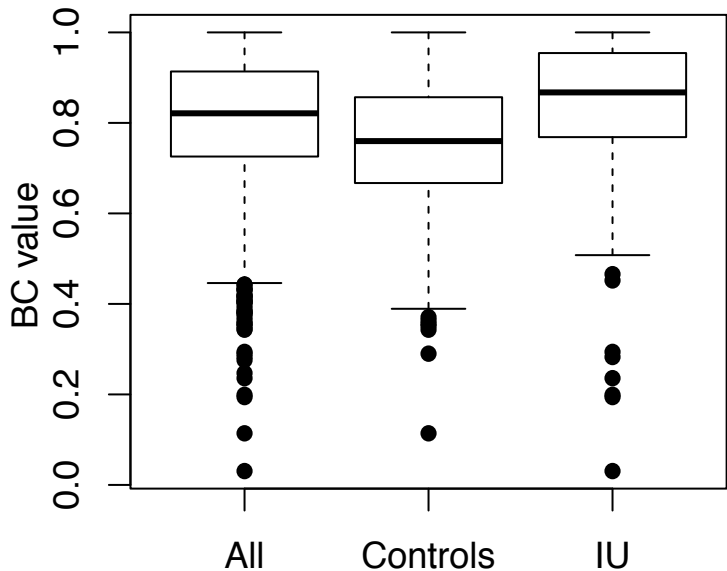

Supplement: S3 Fig — Bray-Curtis dissimilarity for 46 Controls and 39 IU patients. (PDF) [file pone.0201380.s003.pdf]

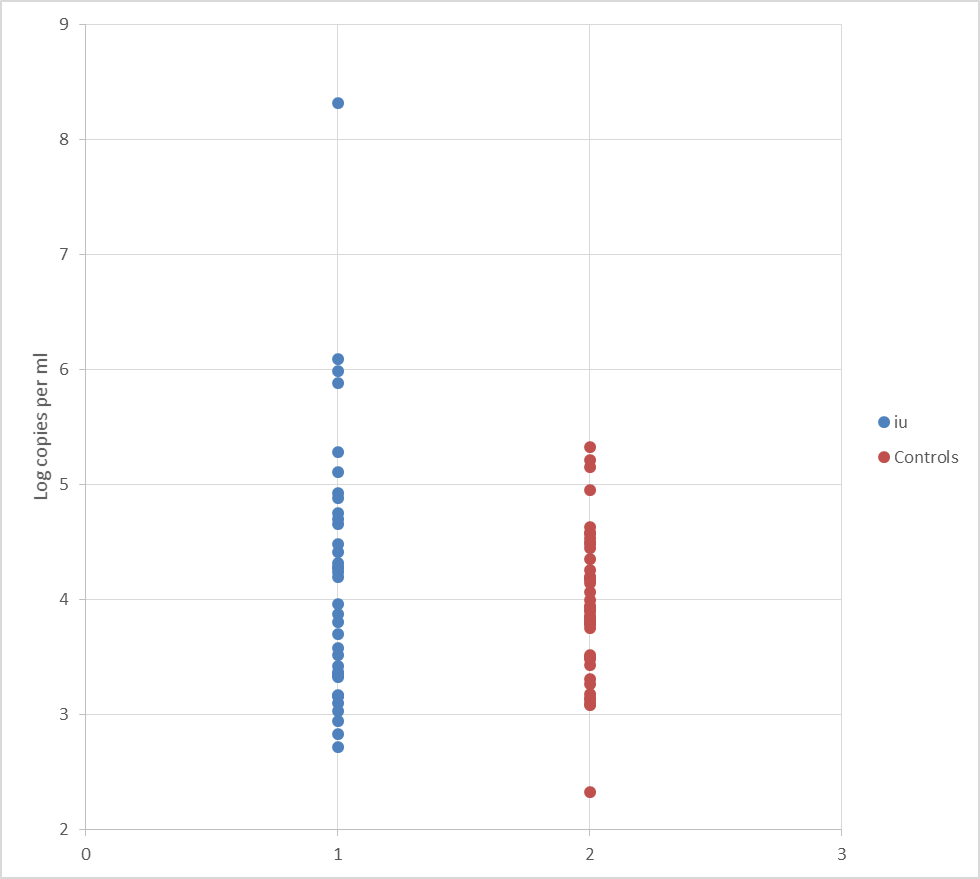

Supplement: S4 Fig — Bacterial load in IU patients and controls as determined by qPCR. (TIF) [file pone.0201380.s004.tif]
